# Effects of a remotely supervised physical training program combined with cognitive training for older individuals at increased risk of clinical-functional vulnerability: study protocol for a randomized clinical trial

**DOI:** 10.1186/s13063-023-07567-8

**Published:** 2023-08-21

**Authors:** Franciele Costa Berní, Ana Carolina Kanitz, Camila Miranda, Dener Budziarek de Oliveira, Marco Bergamin, Valentina Bullo, Gustavo Zaccaria Schaun, Cristine Lima Alberton

**Affiliations:** 1 Physical Education School, Universidade Federal de Pelotas, Pelotas, RS Brazil; 2 School of Physical Education, Physical Therapy and Dance, Universidade Federal Do Rio Grande Do Sul, Porto Alegre, RS Brazil; 3 Department of Medicine, University of Padova, Padua, Italy; 4 Centre for Sport Science and University Sports, University of Vienna, Vienna, Austria

**Keywords:** Exercise, Physical training, Cognitive training, Tele-rehabilitation, Elderly

## Abstract

**Background:**

Despite the robust body of evidence for the benefits of home-based physical exercise, there is still a paucity of data on the benefits of home-based cognitive training for older adults, especially in those at increased risk of clinical-functional vulnerability. As such, the present study aims to compare the chronic effects of a telehealth-delivered physical training intervention alone or combined with a cognitive training program in older adults at increased clinical-functional vulnerability risk.

**Methods:**

A randomized clinical trial will be conducted including 62 sedentary older individuals classified as at increased risk of clinical-functional vulnerability based on their Clinical-Functional Vulnerability Index score. Participants will be randomly allocated in a 1:1 ratio to one of two groups, an intervention group including physical training combined with cognitive training, or an active control group including physical training alone. Both groups will receive home-based supervised training remotely for 12 weeks and will be assessed for the primary and secondary outcomes of the study before and after the training period. Primary outcomes include cognitive function and dynamic balance with a dual task. Secondary outcomes encompass physical, cognitive, and occupational performance, functional capacity, quality of life, and anxiety and depression symptoms, as well as hemodynamic measures. Data analysis will be performed by intention-to-treat and per protocol using mixed linear models and Bonferroni’s post hoc (*α* = 0.05).

**Discussion:**

Our conceptual hypothesis is that both groups will show improvements in the primary and secondary outcomes. Nevertheless, we expect physical combined with cognitive training to improve cognitive function, dual task, and occupational performance to a greater degree as compared to physical training alone.

**Trial registration:**

NCT05309278. Registered on April 4, 2022.

**Supplementary Information:**

The online version contains supplementary material available at 10.1186/s13063-023-07567-8.

## Background

The aging process is a natural and multifactorial phenomenon that involves the complex interaction between biological and molecular mechanisms. This interaction may differ between individuals, but overall, reductions in autonomy and independence are observed as people age [51]. As a consequence of these losses, there is a progressive increase in older adults’ social, physical, and cognitive vulnerability [44], with a marked raise on the incidence of diseases and the risk of physical disability, dependency, and mortality in those objectively classified as being at an increased risk of clinical-functional vulnerability [20]. In this sense, the latter covers multidimensional aspects of older individuals’ health, encompassing domains related to functional capacity and mobility, and also cognition, communication, self-perceived health, among others [35].

Physical exercise is an ally for improving the health of older individuals at increased risk of clinical-functional vulnerability, which has been evidenced as a key non-pharmacological strategy towards this goal [33]. Indeed, a reduction in the physical activity levels of older adults is strongly associated with increased cardiovascular risk, social isolation, frailty, sarcopenia, cancer, diabetes, and other metabolic diseases [10]. Regular physical exercise, on the other hand, brings about several benefits in physical fitness and functional capacity, such as increases in muscle mass, strength, power, agility, balance, maximal oxygen uptake, and independence in carrying out activities of daily living [24, 26, 31, 47, 50].

Meanwhile, cognitive impairments are also responsible for the increased clinical-functional vulnerability risk in some older adults. In these individuals, exposure not only to physical exercise but also to cognitive exercises might be advantageous [4, 28, 45]. In fact, aging is linked to structural changes in some regions of the brain, such as the frontal and medial temporal lobes, which gradually impair older people’s capacity to solve new problems [58]. Cognitive training, therefore, aims at improving different cognitive functions such as memory, language, attention, and processing speed, as well as depression, possibly delaying age-related cognitive decline [18, 42]. A previous randomized controlled trial analyzed the effect of cognitive training in 2832 older individuals [42]. The authors observed improvements in aspects related to memory, reasoning, and processing speed, coupled with lower self-reported difficulty in performing instrumental activities of daily living, ultimately leading to a lower cognitive decline and greater independency levels compared to the control group.

A risk factor that can also influence the physical and cognitive capacity of older individuals is social isolation [37, 52]. Recently, the world population was forced to shelter at home as a consequence of several lockdown measures to reduce the transmission of the COVID-19 virus. Such measures, although necessary, were not without a price [2]. Several reports now indicate that lockdown measures negatively impacted individuals’ physical and cognitive well-being [8, 49], and increased loneliness [19], especially in at-risk populations such as older adults. In the latter, social isolation typically leads to an increased risk of cardiovascular and autoimmune diseases, functional impairment, and neurocognitive and mental health problems [7], as well as anxiety and depression symptoms [46]. Because the restrictions imposed by the pandemic prevented these individuals from attending commonly sought-after activities to improve their physical and cognitive well-being, such as going to a gym, for example, alternatives needed to be found.

Telehealth delivered exercise training was one such alternative [17]. Specifically, the use of remotely supervised home-based physical exercise programs delivered via telehealth as a safe strategy to deliver training interventions during the COVID-19 pandemic proved successful [15]. A recent randomized controlled trial tested the effectiveness of this approach and concluded that a synchronous telehealth-delivered exercise program improved physical fitness, quality of life, and mood in older adults [3]. As a result, home-based online training is expected to remain a viable exercise tool even after the pandemic is over [5].

Despite the current body of evidence on the benefits of home-based physical exercise, there is still a paucity of data on the benefits of home-based physical exercise combined with cognitive training for older individuals, especially those at greater risk of clinical-functional vulnerability. As such, the present study aims to investigate the effects of a telehealth-delivered physical training intervention combined with cognitive training as compared to physical training alone in older adults at increased risk of clinical-functional vulnerability. Herein, we will report the protocol of the REPHYCOVE Study. We hypothesized that combined physical and cognitive training would result in additional positive effects for the studied population, compared to physical training alone, on cognitive function, dual task, and occupational performance.

## Methods

### Trial design

The REPHYCOVE Study is a randomized, single-blind, parallel, controlled, superiority trial. Participants are randomly allocated in a 1:1 ratio to one of two groups: an intervention group including physical training in combination with cognitive training (PCT) or an active control group including physical training alone (PT). The groups receive supervised training remotely for 12 weeks and are assessed for the primary and secondary outcomes of the study before and after the training period. The present study protocol follows as closely as possible the SPIRIT Statement recommendations [27] and was previously registered in the Clinical Trials database (NCT05309278).

### Study setting

This trial is being conducted at the Physical Education School of the Universidade Federal de Pelotas (Brazil).

### Eligibility criteria

The inclusion and exclusion criteria for participants are defined as follows:

Inclusion criteria.

1. 60 years of age or older (both sexes),
2. Mini-Mental State Examination (MMSE) score equal to or greater than 19 points,
3. Elementary school education complete or over,
4. Sedentary (no current or previous participation in structured exercise > 1x/week in the past 6 months),
5. Increased risk of clinical-functional vulnerability, as determined by the Clinical-Functional Vulnerability Index-20 (CFVI-20) questionnaire (score ≥ 7),
6. Access to a cell phone, tablet, or notebook with internet access,
7. Resident in the city of Pelotas, Brazil.

Exclusion criteria.

1. Individuals who have been severely affected by COVID-19,
2. Not retired or those retired individuals who maintained continuous or sporadic work activities,
3. Neuromuscular deficits or any medical diagnosis that prevents the individuals from performing physical exercises,
4. Individuals with decompensated or untreated blood pressure (> 140 × 90 mmHg) and,
5. Individuals with visual problems that prevent them from watching the training sessions on their cell phone, tablet or notebook screen.

### Sample size

The sample size calculation was performed using the GPower v. 3.1 software, adopting a significance level of *α* = 0.05 and a power of 80%. Data used in the determination of the effect size *f* for each primary outcome (cognitive capacity: *f* = 0.35; dynamic balance: *f* = 0.22; dual-task performance: *f* = 0.20) were extracted from [16, 29], resulting in a total sample size of 52 participants. To account for possible losses during the study procedures, ten additional participants (i.e., ~ 20%) are to be recruited for the study, totaling 62 participants randomized between the two groups.

### Recruitment

The recruitment period began in July 2022 and is still ongoing. Participants are recruited voluntarily through notes published in local or regional newspapers, invitations posted on social media, and advertisements posted at several Basic Health Units in the city of Pelotas, Brazil. Those individuals who respond to our advertisement are contacted by two study team members (F.C.B and D.B.O). They are asked key questions related to age, working activities, and whether the person had internet access as an initial filter before directing the individual to the second phase of recruitment. Whenever the previous criteria are fulfilled, the investigators apply an anamnesis, the MMSE and the CFVI-20 questionnaires online through the Google Meet platform to verify if the participant meet the remainder of the inclusion criteria (see above). Those deemed eligible are invited to read and sign an online informed consent form on the Google Forms® platform containing detailed information about the study, which is followed by the schedule of a baseline measurements session (herein defined as weeks − 1 and 0).

### Randomization, assignment of interventions, and blinding

Participants included in the study are first stratified based on their MMSE score and then randomized in a 1:1 ratio between the two groups using blocks of different sizes. Randomization is performed on the www.randomization.com website by an investigator (C.M.) not involved with the assessments and training of the participants after the baseline measurements are completed. The same investigator is responsible for contacting each participant by telephone to inform which group he/she is allocated to and provide detailed information on the days and time that the online training sessions will occur. Blinding is applied to the primary and secondary outcome assessors at baseline, and a similar procedure will also be applied at the post-intervention assessment procedures. Due to the nature of the interventions, the team conducting the exercise sessions and the participants performing them cannot be blinded.

### Interventions

The training sessions are conducted in small groups ranging from 3 to 7 participants by the same investigators throughout the study. A detailed description of each intervention is provided below:

#### Physical training only group

The PT group acts as an active control group that only perform the physical training intervention, which is presented in detail in Table 1. Specifically, participants assigned to this group receive two remotely supervised physical training sessions per week for a total of 12 weeks. Each session begins with a 5-min general warm-up and end with a 5-min stretching, whereas the main part has 20 min during the first training mesocycle (week 1) and progresses to up to 35 min at the end of the intervention (weeks 9–12). The exercises are performed in a circuit fashion at an intensity corresponding to 3 on Borg’s CR10 rating of perceived effort scale (i.e., moderate). Participants will be asked to arrange the space in which the exercise sessions are to be performed (e.g., their living room) with safety as a priority, ensuring the absence of any objects that may pose a risk of falling. Participants will also receive clear instructions to stay near a fixed structure or a chair, allowing them to stabilize themselves if they experience a sense of imbalance or the need to rest during the exercise sessions.

**Table 1:** Physical training program periodization

Week	Exercise	Observations	Sets	Reps/duration	Rest between sets	Intensity
1	Single-leg balance	W/support	1	30″	-	3^a^
	Guided chair squat		1	10x		
	Wall push-ups		1	10x		
	Wall squat	Isometric	1	15″		
	Lateral shoulder raise		1	10x		
	Standing hip abduction	W/support	1	10x		
	Bent over row		1	10x		
	Plantar flexion		1	10x		
	Seated leg raise	Isometric	1	30″		
2–4	Single-leg balance	W/support	2	35″	2′	3
	Guided chair squat		2	12x		
	Wall push-ups		2	12x		
	Wall squat	Isometric	2	20″		
	Lateral shoulder raise		2	12x		
	Standing hip abduction	W/support	2	12x		
	Bent over row		2	12x		
	Plantar flexion		2	12x		
	Seated leg raise	Isometric	2	35″		
5–8	Single-leg balance	W/support	3	30″	2′	3
	Guided chair squat		3	10x		
	Sofa push-ups		3	10x		
	Wall squat	Isometric	3	15″		
	Lateral shoulder raise		3	10x		
	Standing hip abduction		3	10x		
	Bent over row		3	10x		
	Plantar flexion		3	10x		
	Seated leg raise	Isometric	3	30″		
9–12	Single-leg balance	W/support	3	35″	2′	3
	Free squat		3	12x		
	Sofa push-ups		3	12x		
	Wall squat	Isometric	3	20″		
	Lateral shoulder raise		3	12x		
	Standing hip abduction		3	12x		
	Bent over row		3	12x		
	Plantar flexion		3	12x		
	Seated leg raise	Isometric	3	35″		

^a^Borg’s CR10 scale

#### Physical and cognitive training group

The PCT group receives the same physical training intervention as the PT group. However, in addition to the two weekly physical training sessions, this group receives one additional remote cognitive training session per week for a total of 12 weeks, with at least 24 h separating it from the physical training sessions. Specifically, each cognitive training session begins with a 5-min conversation, followed by 45 min of cognitive exercises (described in detail in Table 2), and a 10-min final conversation including instructions for the participants. In addition to the cognitive exercises performed during this session, participants assigned to the PCT group receive a set of cognitive activities to be performed daily asynchronously (described in detail in Table 3), totaling 84 cognitive training sessions at the end of the intervention period.

**Table 2:** Cognitive exercises that will be performed during the weekly cognitive training sessions

Week	Sets	Exercise	Duration
1	2	Write with the non-dominant hand two random sentences, chosen by the investigators	45′
2	3	Each participant must name the greatest number of animals as possible in one minute, which will be registered by the investigators	45′
3	2	Observe a random image and give it at least 5 different adjectives (up to 10)	45′
4	2	With a set of scrambled letters, try to form a word	45′
5	3	Read a word and think of five others that begin with the same letter	45′
6	3	Count from 0 to 100 backwards	45′
7	3	Count from 0 to 100 backwards saying only the even numbers	45′
8	1	Memorize what they need to buy in the market, without making a shopping list	45′
9	2	Read a sentence and make another sentence using the same words	45′
10	3	Each participant must name the greatest number of animals as possible in one minute, which will be registered by the investigators	45′
11	2	Observe a random image and give it at least 5 different adjectives (up to 10)	45′
12	3	Count from 0 to 100 backwards saying only the odd numbers	45′

**Table 3:** Cognitive exercises that will be performed asynchronously throughout the intervention

Week	Daily frequency	Exercise	Duration
1–2	2	Get dressed with his/her eyes closed while seated	Indefinite
3–4	3	Brush his/her teeth using the non-dominant hand	Indefinite
5–6	2	See the time (i.e., clock) in a mirror	Not applicable
7–8	2	Eat using the non-dominant hand	Indefinite
9–10	2	Observe photos of his/her choice, upside down, while analyzing them	Not applicable
11–12	1	Turn off the lights in the house while singing	Indefinite

### Criteria for discontinuing allocated interventions

Participants may be discontinued from the study at any moment if they withdraw their consent to participate or report a lack of interest or unwillingness to continue the trial. In case the participant has already been allocated to any of the study arms, participation in the trial is interrupted if safety concerns such as a disease complication or a severe health event occurs that precludes attendance to the intervention sessions or in the case of medical request or advice.

### Trial retention strategies

Participants assigned to both groups are contacted the day before each training session via a standardized text message to remind them of the time the session is scheduled and to reinforce the importance of their participation in the intervention sessions. Additionally, phone calls or WhatsApp® messages are used to inquire participants about any adverse event that might have happened in the cases where a participant misses a training session without warning. Training sessions in which participants do not attend are not recovered, maintaining intention-to-treat analyses.

### Outcomes

Primary and secondary outcomes are assessed on three separate days, first at baseline (week 0) and then after the training intervention (week 13). Outcomes are assessed for all randomized participants, irrespective of attendance or completion status. Those who withdraw from the study at any time after randomization are also invited to perform the final study evaluations for inclusion in the intention-to-treat analysis.

The first testing day is held online. Participants answer an anamnesis, and the MMSE and IVCF questionnaires are applied. The second testing day also occurs online. During this session questionnaires related to the self-perceived quality of life, anxiety and depressive symptoms, occupational performance, working memory, and cognitive performance are applied individually by the investigators. Due to the personal nature of the data, assistance to complete the questionnaires is only provided by the investigators if completely necessary. On the third day, participants attend an in-person testing session at the Neuromuscular Assessment Laboratory of the Federal University of Pelotas. Dynamic balance, lower limb strength and endurance, handgrip strength, gait speed, and office blood pressure measures are performed. For all testing sessions, participants are instructed not to perform any intense physical activity 72 h before. Further, post-intervention measures are performed 72 h after the last training session. The temporal description of the study procedures is shown in Table 4.

**Table 4:** Temporal description of the study protocol

		**Baseline**		**Intervention**	**Post-intervention**	
**Timepoint**	Week − 1	Week 0 (remotely)	Week 0 (in-person)	Weeks 1–12 (remotely)	Week 13 (remotely)	Week 13 (in-person)
***Enrolment***						
Eligibility screening	X					
Informed consent	X					
Allocation	X					
***Experimental conditions***						
Physical training (control)				X		
Physical and cognitive training				X		
***Assessments***						
*Primary outcome measures*						
MMSE		X				
Dual-task performance			X			X
*Secondary outcome measures*						
CFVI-20		X			X	
Timed Up-and-Go			X		X	X
Self-reported QoL		X			X	
Anxiety and depression		X			X	
Occupational performance		X			X	
Digit span		X			X	
Verbal fluency		X			X	
Handgrip strength			X			X
30-s Sit-to-Stand			X			X
Systemic blood pressure			X			X

*CFVI-20* Clinical-functional vulnerability index, *MMSE* mini-mental state examination, *QoL* Quality of life

The same investigators (F.C.B and D.B.O) are responsible for the application of the tests at both the baseline and post-intervention timepoints. Outcome assessors are trained, and a standard operating procedures manual is available to them during the testing procedures. Of note, outcome assessors and the investigators responsible for analyzing the primary and secondary outcomes listed in this protocol are blinded to the participant’s group.

### Primary outcomes measures

The primary outcomes of this study will focus on changes in cognitive function (as assessed by the MSSE score) and dual-task performance (measured during the TUG test) from baseline to post-intervention. This selection is motivated by the increased fall risk observed in older adults during concurrent tasks, such as walking while engaging in other cognitive or motor activities. Considering that daily life entails a wide range of dual- and multi-tasks, interventions that positively impact cognitive and functional outcomes are imperative for older individuals at a heightened risk of clinical-functional vulnerability [12].

#### Cognitive function

In addition to being an eligibility criterion for participants’ inclusion at baseline, the MMSE score is also used to assess cognitive function responses to the two training interventions investigated [29]. The questionnaire is divided into two sections: (1) the first requires verbal answers related to temporal and spatial orientation, memory, and attention, with a maximal score of 21 points; (2) the second section measures the ability to name objects, follow verbal and written commands, write a sentence spontaneously, and copy a complex polygon; a maximum score of 9 points is attributed to this section. The MMSE total score represents the sum of the scores from the two sections and ranges from 0 to 30 points [4]. Participants’ cognitive function is classified based on the education-adjusted cut-off scores as proposed by Brucki et al. 2003 [14].

#### Dual-task performance

To investigate older adults’ cognitive capacity and possible deficits, such as difficulty dividing attention or switching between tasks [1, 32], the TUG test is applied while simultaneously performing a verbal task to determine the influence of a cognitive task on physical function. Specifically, participants are asked to sit in a chair with a height of ≈ 46 cm, with their backs against the backrest and their hands on their legs. After the researcher’s signal, the participants should get up without using their arms, walk 3 m at their normal pace, turn around, walk back to the chair, and sit down until their backs are in touch with the backrest again [41]. During the procedure, participants must name the maximum number of animals possible, and the total number of animals recalled and the time to complete the test is recorded [16]. In addition, the dual-task cost is also calculated using the following formula: *Dual-task cost* = *[dual-task performance (s) – single-task performance (s) / single-task performance (s)* × *100]*, as previously described by [40].

### Secondary outcomes measurements

In addition to cognitive function and dynamic balance, a set of clinically relevant secondary outcomes for older adults were also established for this study and are described in detail below.

#### Clinical-Functional Vulnerability Index (CFVI-20)

In addition to being an eligibility criterion for participants’ inclusion at baseline, the CFVI-20 score is also used to assess the responses to the two training interventions investigated. This questionnaire covers multidimensional aspects of the older adult’s health condition and is a viable tool for screening older individuals at increased risk of frailty [35]. It is divided into eight sections: age, health self-perception, functional disabilities (three instrumental activities of daily living and one activity of daily living), cognition, mood, mobility (reaching, grasping and pinching, aerobic/muscular capacity, gait, and sphincter continence), communication (vision and hearing), and the presence of comorbidities. There are 20 questions, which result in a maximum of 40 points. The higher the score, the greater the risk of clinical-functional vulnerability [34]. In addition, CFVI cut-off values are also applied to classify the level of clinical-functional vulnerability, with scores ranging 0–6, 7–14, and  ≥ 15 points considered as having a low risk, an increased risk, or an installed risk of clinical-functional vulnerability or frailty, respectively [34]. Older individuals with a score  ≥ 7 will be included in this study.

#### Dynamic balance

The TUG test without a simultaneous cognitive task is applied for measuring dynamic balance. As previously explained, participants are asked to sit in a chair with a height of ≈ 46 cm, with their backs against the backrest and their hands on their legs. After receiving the orientation, the participants should get up without using their arms, walk 3 m at their normal pace, turn around, walk back to the chair, and sit down until their backs are in touch with the backrest again [41]. Those older adults who can perform the test in 10 to 20 s are considered independent. However, those who take longer than 20 s to complete the test are possibly in a state of postural instability and at higher risk for falls [38].

#### Handgrip strength

The amount of force produced by a maximal isometric contraction of the extrinsic hand muscles of the dominant upper limb is measured using a hand dynamometer, and handgrip strength is recorded in kgf. The test is performed with the participant seated, with his or her shoulder in a neutral position close to the body and the elbow flexed at 90° without support. Three attempts are provided, and the average value is considered for analysis [23].

#### 30-s Sit-to-Stand test

Lower limb strength is assessed using the 30-s Sit-to-Stand test, which involves counting the number of times the participant can stand up entirely from the sitting position with arms crossed over the chest. The performance is measured based on the total number of times the participant can perform the movement for 30 s [43].

#### World Health Organization Quality of Life-bref (WHOQOL-bref)

The self-reported quality of life is assessed by the WHOQOL-bref questionnaire. It is a 26-item questionnaire covering four domains: physical health, psychological health, social relationships, and environment [54]. The questions must be answered using a Likert scale ranging from 1 (e.g., very dissatisfied) to 5 (e.g., very satisfied). Domain scores for the WHOQOL-BREF are calculated by multiplying the mean of all items in each domain by a factor of four. These scores are then transformed to a 0–100 scale as described in detail elsewhere [56], and higher scores indicate a better self-perceived quality of life [44].

#### Hospital Anxiety and Depression Scale (HADS)

The HADS is an instrument composed of 14 items, seven of which are related to anxiety and seven related to depression, which allows the assessment of symptoms in the previous week. Each HADS item ranges from 0 to 3 points, for a maximum of 21 points in each subscale (i.e., anxiety and depression). This instrument was developed by Zigmond and Snaith [59] and was previously translated and validated for the Brazilian population [13].

#### Canadian Occupational Performance Measure (COPM)

The COPM is a semi-structured interview that uses a typical day as a reference to identify issues in self-care, productivity, and leisure based on the self-reported performance capacity and satisfaction of the patient in occupations the participant needs, wants, and/or is expected to do. Initially, the participant is asked to list the main occupational difficulties he or she encounters in the areas of leisure, productivity, and self-care. The participant then is asked to score each of the issues mentioned from 1 (least important) to 10 (most important). Based on the participant’s list, the five most important occupational performance issues are selected so that the participant can classify their performance and their satisfaction in each specific activity, again using a scale from 1 to 10. The performance scores are summed and divided by the number of issues reported, and the same is done with the satisfaction scores to obtain the COPM result [39].

#### Digit span test (DST)

The DST is used to evaluate working memory. During the test, participants are asked to verbally recall a series of numbers that are said by the researcher. The test begins with three numbers from 0 to 9, which are read with a 1 s interval between each number, and that the participant should recall in the correct order. In case the participant recalls them correctly, a new sequence is provided with an additional digit (i.e., four digits), and so on. The test ends when the participant is not able to recall the correct sequence two times in a row. The higher the number of digits recalled, the better the working memory [48].

#### Verbal fluency test

Participants must name as many animals as they know in 1 min, controlled by the assigned researcher using a stopwatch. Different species and animals that have different names for their male and female versions (e.g., ox and cow) are only counted once. The score is considered the total number of animals recalled during the assigned time and classified according to education-adjusted cut-off points [36]. Because both the Verbal Fluency and Dual-task tests require participants to name different animals, the tests will be performed on separate days to avoid or reduce potential interference.

#### Systemic blood pressure

Resting systolic (SBP) and diastolic (DBP) blood pressure measurements are taken in the sitting position using an automated blood pressure monitor (Omron, HEM-7200, China) according to established guidelines [55]. One measurement is taken on each arm with a 1-min interval, and two additional measurements are taken on the arm that shows the highest blood pressure value. The mean of the three SBP, DBP, and resting HR measurements performed on the same arm will be used for the analysis.

### Medical authorizations

Medical authorizations for the practice of physical exercises are requested for each randomized participant. In addition, adverse events are assessed through the intervention and classified according to severity (mild, moderate, or severe), predictability (expected or unexpected), and potential relationship to the study procedures (definitely related, possibly related, or unrelated). All adverse events are discussed by at least two of the following members of the study team before a consensus is reached: main researcher (C.L.A), study manager (F.C.B), and additional medical consultants and experts.

### Adherence assessments

Adherence measures encompass both attendance and compliance of interventions by the randomized participants. Attendance is monitored based on the frequency of sessions participants took part in and is reported as the percent of sessions experienced by each participant, given the total number of scheduled sessions. Compliance, on the other hand, is assessed as the percentage of training sessions performed without significant protocol deviations.

### Data management

All outcome measures are collected through online forms, identified by participant number ID. Each form contains instructions related to standard operating procedures, and a specific researcher is responsible for checking missing or inaccurate data. At the end of each testing day, the same researcher is responsible for creating a backup of the data, which are stored in a cloud system and additionally stored on an external hard drive for security purposes.

### Data monitoring and auditing

The present study does not have a data monitoring committee due to the limited availability of resources. Despite its high value for the overall quality of the study, such a committee would not be mandatory due to the characteristics of the types of interventions and outcomes included in the REPHYCOVE Study.

### Statistical analysis

Analyses will be performed using the SPSS software v. 25.0, adopting an *α* = 0.05. Descriptive data will be presented as means, standard deviations, 95% confidence intervals, or absolute and relative frequencies. Data normality will be tested by the Shapiro–Wilk test, and the Levene test will test the homogeneity of variances. Primary and secondary outcome analyses will be conducted using mixed linear models with “treatment group x timepoint” interaction terms and using baseline values as covariates. An intention-to-treat analysis will include all randomized participants, in which missing data will be imputed using multiple imputations. In contrast, a per-protocol analysis will be performed with only those participants with an attendance rate  ≥ 80%. Effect sizes between-group will be computed based on Cohen’s *d* and the 95% confidence interval.

### Ethics and dissemination

#### Ethics approval and consent to participate

The study protocol was approved by the Universidade Federal de Pelotas Ethics Review Board (n. 5.471.834). Specific COVID-19 safety protocols are being followed based on the [21]. Individuals are informed about all procedures related to the study participation, including possible risks and benefits, and sign an online informed consent form on the Google Forms platform. The responsible researcher (F.C.B) is available to answer any questions related to the procedures as needed. If necessary, amendments to the study protocol will be communicated to the Ethics Review Board, and our research team will update the protocol of the clinical trial registry accordingly.

#### Confidentiality

Participants’ identities are preserved, and their respective data are identified using individual identifying numbers (ID).

#### Conflict of interest

The authors declare no competing interests.

#### Dissemination policy

Upon completion of the study, our dissemination plan aims to circulate the study’s results to as many interested parties as possible. First, participants will receive individualized reports with their results in a language comprehensible to the lay public. Participants will also receive general guidance on how to maintain the practice of physical and cognitive exercises, as well as information on successful aging and general health care. Additionally, the study’s main findings will also be published in the press for the general public. Finally, scientific dissemination will be achieved through the publication of research manuscripts and posters and/or oral presentations at scientific events.

## Discussion

According to the 2022 World Population Prospects [57], it is estimated that by 2050 one in every six people in the world will be over 65 years old, compared to one in every ten individuals in 2022. As a consequence of this, our society will undergo a comprehensive demographic transition. Thus, it is not only essential to understand how the aging process happens but also how one can intervene to improve longevity and the health span of this already considerable portion of the world’s population. To achieve this goal, access to non-pharmacological strategies such as physical and cognitive exercise training will be of absolute importance [34].

The COVID-19 pandemic, which began in 2019, directly and indirectly affected the health of millions of people around the globe [9]. Several reports indicate that the lockdown measures taken to contain the spread of the virus, although necessary, negatively impacted the physical and cognitive well-being of many of these individuals [8, 49]. In older people who were followed from December 2020 until August 2021, a significant decline in physical and cognitive function, and a worsening in self-perceived quality of life and the incidence of depressive symptoms was observed [9]. As a consequence, this population not only had to deal with aspects related to aging, but also with negative aspects associated with the pandemic.

The regular practice of physical exercises has been shown as a key factor towards the maintenance of older adults’ functional capacity, physical fitness, strength, agility, balance, muscle quality, cognitive capacity, quality of life, reduction of depressive symptoms, and independence in carrying out activities of daily activities [25, 26, 31, 47]. The combination of such an exercise program with the regular practice of cognitive exercises has been further shown as relevant for those at an increased risk of frailty. Specifically, improvements in physical and cognitive domains have been observed, such as self-perceived quality of life, gait speed, strength, balance, global cognitive function, verbal fluency, memory, executive function, inhibitory control, mood, among others [4, 30, 45, 50].

Remote exercise emerges as a relevant tool for situations where individuals cannot attend in-person activities. Even in people with Parkinson’s disease, remote training has been shown as a safe and feasible alternative to implementing complex training programs [53], with only 11 adverse events being reported in response to 6 months of intervention in 130 participants, seven in the intervention group, and four in the active control group. Similar results were also observed in older adults and in fibromyalgia patients [3, 22]. Overall, these studies demonstrate the effectiveness of remote training intervention for older and at risk populations. They also corroborate the aims of the present study and especially the importance of staying active during situations of social isolation or when some other aspect prevents the performance of in-person exercise (e.g., physical frailty or functional impairments) [6, 11, 17]. Within this context, the present study seeks to take the understanding of remote exercise one step further by combining the established effectiveness of remote physical exercise with the inclusion of a remote cognitive exercise training portion as well, which remains poorly understood.

## Trial status

Protocol version number: May 12, 2023

First day of recruitment: July 26, 2022

Expected end of the intervention: December 31, 2023

### Supplementary Information

**Additional file 1.**

## Data Availability

The datasets used and/or analyzed during the current study will be available from the corresponding author on reasonable request.
